# An Apoptotic Caspase Network Safeguards Cell Death Induction in Pyroptotic Macrophages

**DOI:** 10.1016/j.celrep.2020.107959

**Published:** 2020-07-28

**Authors:** Nathalia Moraes de Vasconcelos, Nina Van Opdenbosch, Hanne Van Gorp, Rosa Martín-Pérez, Annalisa Zecchin, Peter Vandenabeele, Mohamed Lamkanfi

**Affiliations:** 1 Department of Internal Medicine and Pediatrics, Ghent University, 9052 Ghent, Belgium; 2 VIB Center for Inflammation Research, 9052 Ghent, Belgium; 3 Janssen Immunosciences, World Without Disease Accelerator, Pharmaceutical Companies of Johnson & Johnson, Beerse 2340, Belgium; 4 Department of Biomedical Molecular Biology, Ghent University, Ghent 9052, Belgium; 5 Methusalem program, Ghent University, Ghent 9052, Belgium

**Keywords:** pyroptosis, apoptosis, caspase-1, GSDMD, inflammasome, caspase-8, caspase-7, caspase-3

## Abstract

Pyroptosis has emerged as a key mechanism by which inflammasomes promote host defense against microbial pathogens and sterile inflammation. Gasdermin D (GSDMD)-mediated cell lysis is a hallmark of pyroptosis, but our understanding of cell death signaling during pyroptosis is fragmented. Here, we show that independently of GSDMD-mediated plasma membrane permeabilization, inflammasome receptors engage caspase-1 and caspase-8, both of which redundantly promote activation of apoptotic executioner caspase-3 and caspase-7 in pyroptotic macrophages. Impaired GSDMD pore formation downstream of caspase-1 and caspase-8 activation suffices to unmask the apoptotic phenotype of pyroptotic macrophages. Combined inactivation of initiator caspase-1 and caspase-8, or executioner caspase-3 and caspase-7, is required to abolish inflammasome-induced DEVDase activity during pyroptosis and in apoptotic *Gsdmd*^−/−^ cells. Collectively, these results unveil a robust apoptotic caspase network that is activated in parallel to GSDMD-mediated plasma membrane permeabilization and safeguards cell death induction in pyroptotic macrophages.

## Highlights

• Wild-type macrophages display caspase-3/7-dependent DEVDase activity during pyroptosis
• GSDMD inhibition unmasks an apoptotic cascade downstream of inflammasomes
• Inflammasomes trigger apoptosis by distinct mechanisms in GSDMD^−/−^ and Casp1^−/−^ cells
• Caspase-1 and caspase-8 activate caspase-3 and caspase-7 to induce apoptosis in GSDMD^−/−^ macrophages

## Graphical Abstract

**Figure:**
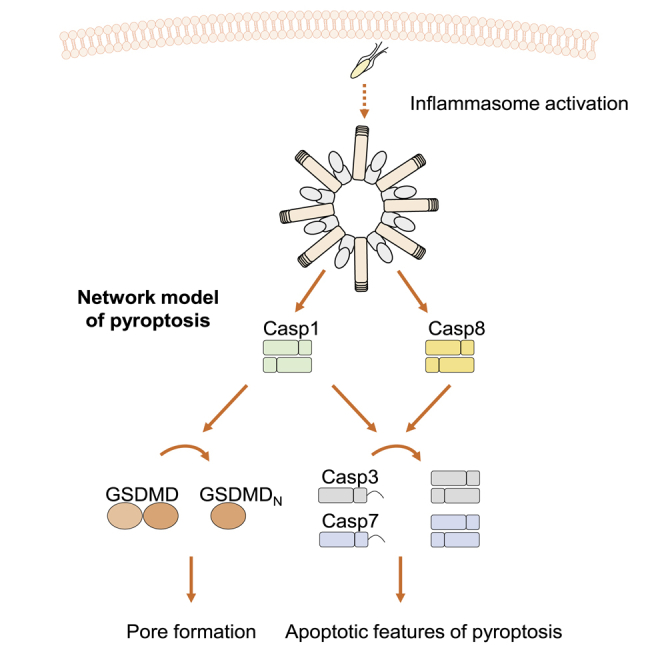

## Teaser

de Vasconcelos et al. demonstrate that an apoptotic cascade is initiated in macrophages undergoing inflammasome-mediated pyroptosis. Caspase-1 and caspase-8 initiate the downstream activation of apoptotic caspase-3 and caspase-7, leading to cleavage of apoptotic markers during pyroptosis. This caspase network explains why inflammasome-activated GSDMD^−/−^ and Casp1^−/−^ cells undergo apoptosis.

## Introduction

Pyroptosis is initiated downstream of inflammasome assembly in activated innate immune cells (Broz and Dixit, 2016; Lamkanfi and Dixit, 2014). It has emerged as a powerful defense mechanism of the host against microbial pathogens (de Vasconcelos and Lamkanfi, 2020). It also drives detrimental autoinflammation, sepsis, and non-alcoholic steatohepatitis (NASH) by promoting passive secretion of interleukin-1β (IL-1β) and alarmins (Kanneganti et al., 2018; Xiao et al., 2018; Xu et al., 2018). Pyroptosis induction by inflammasomes is considered a linear pathway in which murine inflammatory caspase-1 and caspase-11 and human caspase-1, caspase-4, and caspase-5 cleave gasdermin D (GSDMD) to release the N-terminal GSDMD_N_ domain that forms higher-order oligomeric pores in the plasma membrane to induce osmotic swelling and early cell lysis (Aglietti et al., 2016; Ding et al., 2016; Kayagaki et al., 2015; Liu et al., 2016; Sborgi et al., 2016; Shi et al., 2015). This is in marked contrast to apoptosis, in which parallel maturation of apoptotic executioner caspase-3 and caspase-7 by initiator caspase-8 and caspase-9 results in cleavage of hundreds of substrates that orchestrates the coordinated disassembly of the cell without spilling the intracellular content in the extracellular environment (Nagata and Tanaka, 2017).

A wealth of recent findings suggests extensive cross-talk between inflammatory and apoptotic caspases (de Vasconcelos and Lamkanfi, 2020; Fritsch et al., 2019; Newton et al., 2019; Van Opdenbosch and Lamkanfi, 2019). We and others previously demonstrated that apoptosis-associated speck-like protein containing a CARD (ASC) specks serve as cytosolic scaffolds for inflammasome-mediated caspase-8 activation and induction of apoptosis in caspase-1-deficient macrophages in response to stimuli of the Nlrc4, Nlrp1b, AIM2, or Nlrp3 inflammasome pathways (Lee et al., 2018; Pierini et al., 2012; Puri et al., 2012; Sagulenko et al., 2013; Van Opdenbosch et al., 2017). Moreover, an early study showed that caspase-1 activates the apoptotic executioner caspase-7 in wild-type macrophages in response to stimuli of the Nlrp3 and Nlrc4 inflammasomes (Lamkanfi et al., 2008). Yet, the molecular mechanisms in inflammasome-activated macrophages that regulate the switch from pyroptosis to apoptosis signaling remains unclear.

Here, we demonstrate that pyroptosis induced by the Nlrp1b, Nlrc4, and Nlrp3 inflammasomes in wild-type macrophages exhibits hallmark apoptotic features, including activation of apoptotic caspase-3 and caspase-7, DEVDase activity, and cleavage of apoptotic substrates. We show that inflammasome receptors independently engage caspase-1 and caspase-8, both of which redundantly promoted activation of apoptotic executioner caspase-3 and caspase-7 in parallel to GSDMD-mediated plasma membrane permeabilization in wild-type macrophages. Combined inactivation of initiator caspase-1 and caspase-8, or executioner caspase-3 and caspase-7, was required to abolish inflammasome-induced DEVDase activity during pyroptosis as well as in apoptotic *Gsdmd*^−/−^ cells. Notably, impaired GSDMD pore formation downstream of caspase-1 and caspase-8 activation sufficed to unmask the apoptotic phenotype of pyroptotic macrophages. Collectively, these results unveil a robust apoptotic caspase network that is activated in parallel to GSDMD-mediated plasma membrane permeabilization to safeguard cell death induction in pyroptotic macrophages.

## Results

### DEVDase Activity and Cleavage of Apoptotic Substrates during Pyroptosis

**Figure 1:**
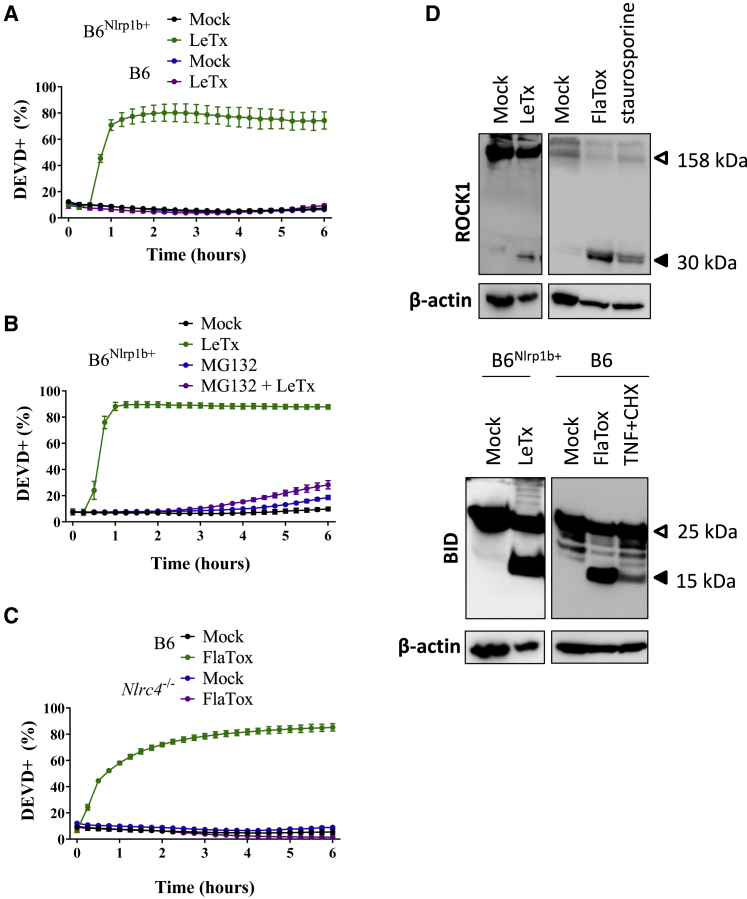
Pyroptosis Features a Caspase-3/7 Signature (A and C) Macrophages of the indicated genotypes were left untreated or stimulated with LeTx (A) or FlaTox (C) in media containing the caspase-3/7 activity (DEVD) probe and imaged on an Incucyte platform. (B) Macrophages of the indicated genotypes were left untreated or pretreated with MG132 (10 μM) for 30 min prior to being stimulated with LeTx in media containing the DEVD probe. Cells were imaged over time on an Incucyte platform. (D) B6^Nlrp1b+^ macrophages (upper panel) or macrophages of the indicated genotypes (lower panel) were treated with LeTx, FlaTox, staurosporine, or TNF+CHX for 2 h, and cell lysates were immunoblotted for the indicated proteins. Results from Incucyte experiments are plotted as the number of positive cells relative to a PI-stained, Triton-x100-treated well (considered 100%). Values represent mean ± SD of technical duplicates of a representative experiment from three biological repeats.

Although peptide substrates may lack selectivity for individual caspases, DEVDase activity is considered a hallmark of caspase-3 and caspase-7 activity that precedes secondary necrosis in cultured apoptotic cells (Aftab et al., 2014). Unexpectedly, we detected a marked increase in DEVDase-positive cells upon induction of pyroptosis in bone-marrow-derived macrophages (BMDMs) of C57BL/6J (B6) mice that hemizygously express a *Bacillus anthracis* lethal toxin (LeTx)-sensitive *Nlrp1b* allele (B6^Nlrp1b+^) (Figure 1A). A steep increase in DEVDase activity occurred ∼90 min after LeTx intoxication and coincided with the pyroptotic plasma membrane permeabilization-associated increase in propidium iodide (PI) fluorescence intensity (Figures 1A and S1A). As control setups, we observed a gradual increase in DEVDase activity over time that mirrored PI positivity when we induced apoptosis in staurosporine-treated B6^Nlrp1b+^ macrophages (Figures S1B and S1C). As expected (Boyden and Dietrich, 2006; Van Opdenbosch et al., 2014; Van Opdenbosch et al., 2017), LeTx-challenged B6 BMDMs remained PI negative (Figure S1A), confirming that a functional *Nlrp1b* allele is required for LeTx-induced cell lysis. LeTx-intoxicated B6 macrophages were also devoid of DEVDase activity (Figure 1A), demonstrating that a functional *Nlrp1b* allele is also required to promote the LeTx-induced DEVDase response in B6^Nlrp1b+^ macrophages. The Nlrp1b inflammasome uniquely requires proteasomal activity for inducing pyroptosis (Fink et al., 2008). Accordingly, pretreatment with the proteasome inhibitor MG132 prevented LeTx-induced DEVDase activity (Figure 1B) and PI positivity (Figure S1D) in LeTx-intoxicated B6^Nlrp1b+^ macrophages. These results show that Nlrp1b-induced pyroptosis is associated with DEVDase activity.

To assess whether DEVDase activity accompanies pyroptosis induced through additional inflammasome pathways, we stimulated wild-type B6 macrophages with FlaTox, a synthetic fusion of the *Bacillus anthracis* (*B. anthracis*) lethal factor N-terminal region fused to *Legionella pneumophila* flagellin (LFn-FlaA) that selectively activates the Nlrc4 inflammasome when targeted to the cytosol with *B. anthracis* protective antigen (PA) (Van Opdenbosch et al., 2017; von Moltke et al., 2012). Increased DEVDase activity in FlaTox-stimulated B6 BMDMs (Figure 1C) occurred concomitant with plasma membrane permeabilization as measured by PI staining (Figure S1E). Loss of *Nlrc4* abrogated FlaTox-induced DEVDase activity and PI staining (Figures 1C and S1E), demonstrating that DEVDase activity is induced following Nlrc4 activation.

Consistent with Nlrp1b- and Nlrc4-mediated pyroptosis being associated with increased DEVDase activity, western blot analysis confirmed cleavage of well-established apoptosis markers in pyroptotic cell lysates (Figure 1D). Caspase-mediated cleavage of ROCK1 in a 30-kDa fragment renders the protein constitutively active and drives apoptotic membrane blebbing (Coleman et al., 2001; Sebbagh et al., 2001). We observed a ROCK1 cleavage fragment in pyroptotic cell lysates of LeTx- and FlaTox-treated B6^Nlrp1b+^ macrophages that was similarly sized to the ROCK1 cleavage fragment of staurosporine-treated macrophages (Figure 1D). Consistent with published reports (de Vasconcelos et al., 2019; Yu et al., 2014), we also observed substantial proteolytic maturation of the pro-apoptotic Bcl2 protein BID into a fragment that appeared of similar size as the tBID cleavage product in apoptotic tumor necrosis factor α (TNF) + cycloheximide (CHX)-treated macrophages (Figure 1D).

### Caspase-1 and Caspase-8 Redundantly Drive Inflammasome-Induced DEVDase Activity

**Figure 2:**
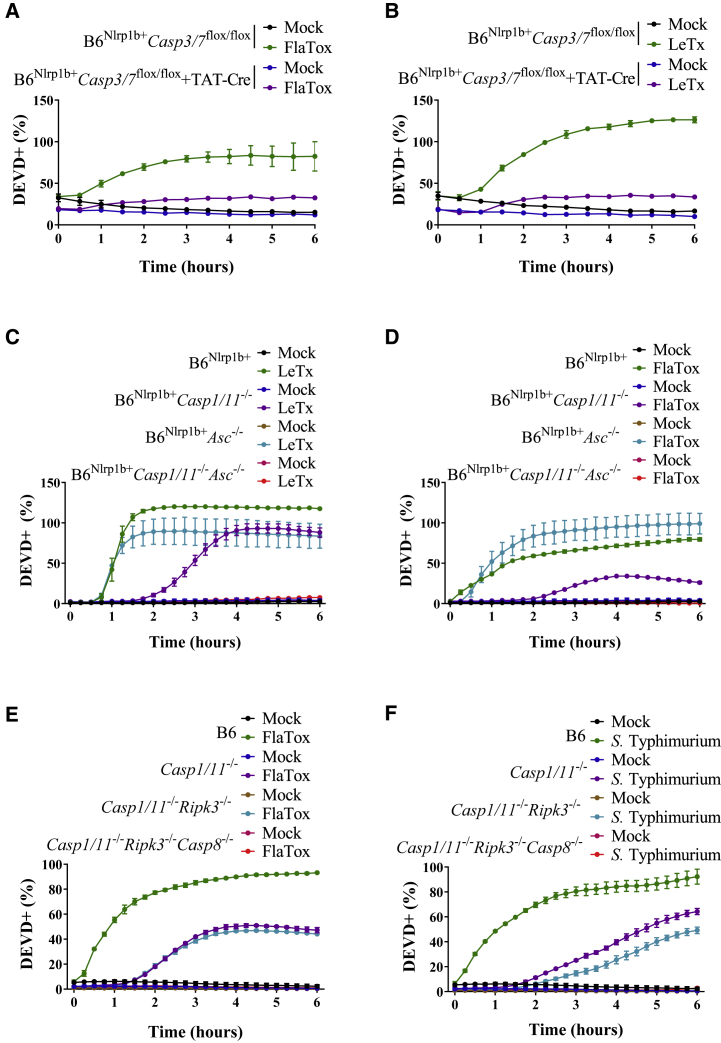
Caspase-1 and Caspase-8 promote Activation of Caspase-3 and Caspase-7 during Pyroptosis (A and B) Macrophages of the indicated genotypes were left untreated or received TAT-Cre (as described in STAR Methods), and subsequently received FlaTox (A) or LeTx (B) or were left untreated in media containing the caspase-3/7 activity (DEVD) probe, and were imaged on an Incucyte platform. (C–F) Macrophages of the indicated genotypes were left untreated or stimulated with LeTx (C), FlaTox (D, E), or log-phase *S.* Typhimurium (F) in media containing the caspase-3/7 activity (DEVD) probe and imaged on an Incucyte platform. Results from Incucyte experiments are plotted as the number of positive cells relative to a PI-stained, Triton-x100-treated well (considered 100%). Values represent mean ± SD of technical duplicates of a representative experiment from three biological repeats.

We next sought to confirm that pyroptosis-associated DEVDase activity genuinely reflects caspase-3/7 activity. Because mice with a combined loss of caspase-3 and caspase-7 are lost shortly after birth (Lakhani et al., 2006), we bred B6^Nlrp1b+^ mice with animals harboring conditionally targeted *Casp3* and *Casp7*alleles (*Casp3/7*^*flox/flox*^) (Saavedra et al., 2018). Downregulated expression of caspase-3 and caspase-7 in BMDMs with cell-permeable active Cre protein (TAT-Cre) (Peitz et al., 2002) relative to untreated B6^Nlrp1b+^*Casp3/7*^*flox/flox*^ BMDMs was confirmed by western blot analysis (Figure S1F). Deletion of these executioner caspases in TAT-Cre-treated B6^Nlrp1b+^*Casp3/7*^*flox/flox*^ BMDMs abolished the induction of DEVDase activity following stimulation with FlaTox (Figure 2A) and LeTx (Figure 2B), confirming that DEVDase activity was driven by activation of executioner caspase-3 and caspase-7 in pyroptotic cells.

To further define the signaling mechanism controlling pyroptotic DEVDase activity, we bred B6^Nlrp1b+^ mice to *Casp1*^−/−^*Casp11*^−/−^ and *Asc*^−/−^ mice to generate B6^Nlrp1b+^*Casp1*^−/−^*Casp11*^−/−^, B6^Nlrp1b+^*Asc*^−/−^ and B6^Nlrp1b+^*Casp1*^−/−^*Casp11*^−/−^*Asc*^−/−^ mice, respectively. We and others previously showed that ASC is dispensable for activation of caspase-1 and induction of pyroptosis by the Nlrc4 and Nlrp1b inflammasomes, whereas it is essential for caspase-8 recruitment and activation (Broz et al., 2010; Lee et al., 2018; Mascarenhas et al., 2017; Pierini et al., 2012; Puri et al., 2012; Sagulenko et al., 2013; Van Opdenbosch et al., 2017). As reported previously (Broz et al., 2010; Guey et al., 2014; Van Opdenbosch et al., 2014), pyroptotic plasma membrane rupture was not impaired in FlaTox- and LeTx-treated B6^Nlrp1b+^ and B6^Nlrp1b+^*Asc*^−/−^ BMDMs (Figures S1G and S1H). Furthermore, DEVDase activity following activation of the Nlrp1b and Nlrc4 inflammasomes continued unabated in B6^Nlrp1b+^*Asc*^−/−^ BMDMs (Figures 2C and 2D), suggesting that caspase-8 is dispensable or may act redundantly with caspase-1 for promoting pyroptotic DEVDase activity. Analysis of B6^Nlrp1b+^*Casp1*^−/−^*Casp11*^−/−^ BMDMs showed that preventing LeTx- and FlaTox-induced caspase-1 activation delays, but it is not sufficient to abolish DEVDase activity and PI staining (Figures 2C, 2D, S1G, and S1H). Contrastingly, combined loss of ASC and caspase-1 fully prevented Nlrc4- and Nlrp1b-induced DEVDase activity and PI staining (Figures 2C, 2D, S1G, and S1H), suggesting that both caspase-1 and caspase-8 mediate inflammasome-induced DEVDase activity. We confirmed (Lee et al., 2018; Mascarenhas et al., 2017; Van Opdenbosch et al., 2017) that LeTx- and FlaTox-induced caspase-8 maturation was abolished in B6^Nlrp1b+^*Casp1*^−/−^*Casp11*^−/−^*Asc*^−/−^ macrophages (Figure S1I), further supporting the notion that caspase-1 and ASC-dependent caspase-8 activation redundantly drive Nlrp1b- and Nlrc4-induced DEVDase activity. To directly test this hypothesis, we established a colony of mice with combined losses in inflammatory caspase-1 and caspase-11 together with caspase-8 in a RIPK3-deficient background to prevent embryonic lethality caused by Casp8 deletion (Kaiser et al., 2011; Oberst et al., 2011). Both DEVDase activity and incorporation of the cell-impermeant cytotoxicity dye Sytox Green were abrogated in BMDMs from these animals that had been stimulated with FlaTox (Figures 2E and S1J). Paralleling these results, *Salmonella enterica* serovar Typhimurium (*S.* Typhimurium) infection failed to mount DEVDase activity and cell lysis in *Casp1*^−/−^*Casp11*^−/−^*Ripk3*^−/−^*Casp8*^−/−^ macrophages (Figures 2F and S1K). Contrastingly, both DEVDase activity and Sytox Green positivity were detected when wild-type BMDMs and macrophages lacking caspase-1 and caspase-11, alone or together with RIPK3, were stimulated with FlaTox (Figures 2E and S1J) or infected with *S.* Typhimurium (Figures 2F and S1K). Collectively, these results imply that caspase-1 and caspase-8 redundantly activate apoptotic executioner caspases in pyroptotic cells parallelly to the induction of GSDMD-mediated plasma membrane permeabilization.

### Impaired GSDMD Pore Formation Downstream of Caspase-1 and Caspase-8 Activation Unmasks Apoptosis

**Figure 3:**
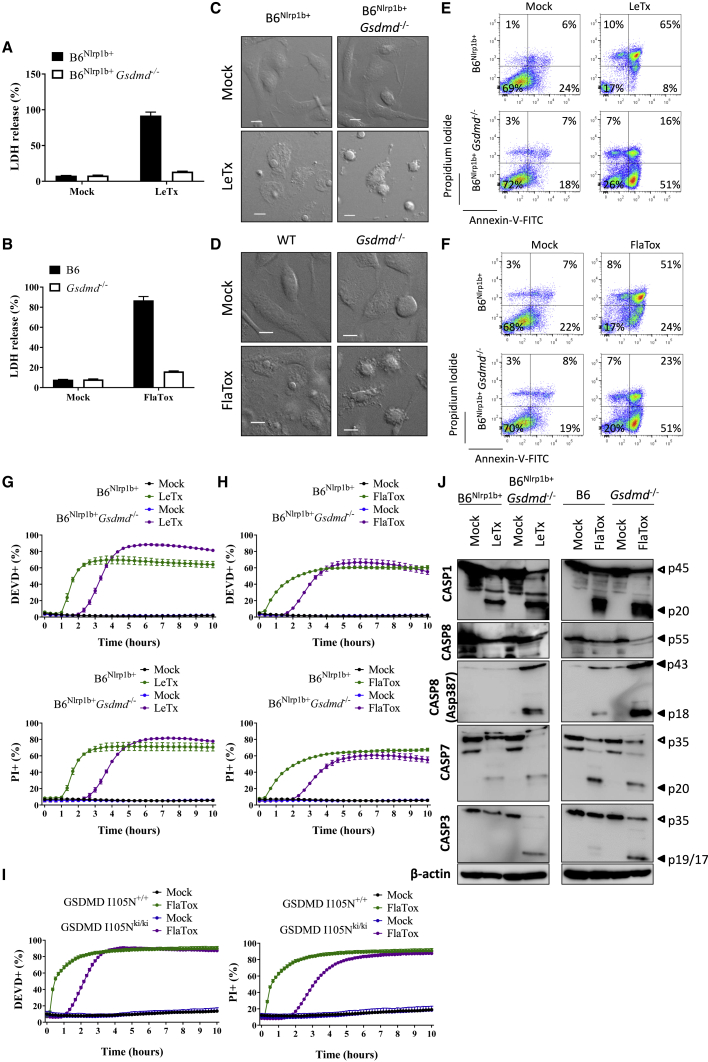
Defective GSDMD Pore Formation Unveils Activation of an Apoptotic Caspase Network by the Nlrp1b and Nlrc4 Inflammasomes (A, C, E, and J) Macrophages of the indicated genotypes were left untreated or stimulated with LeTx for 2 h. Culture supernatants were analyzed for LDH activity (A), cells were imaged under a confocal microscope (C) or analyzed by fluorescence-activated cell sorting (FACS) for Annexin-V/PI positivity (E), and cell lysates were immunoblotted for the indicated proteins (J). (B, D, F, and J) Macrophages of the indicated genotypes were left untreated or stimulated with FlaTox for 2 h. Culture supernatants were analyzed for LDH activity (B), cells were imaged under a confocal microscope (D) or analyzed by FACS for Annexin-V/PI positivity (F), and cell lysates were immunoblotted for the indicated proteins (J). (G and H) Macrophages of the indicated genotypes were left untreated or stimulated with LeTx (G) or FlaTox (H) in media containing DEVD probe and PI and imaged on an Incucyte platform. (I) GSDMD^I105N^ knockin homozygous (GSDMD I105N^ki/ki^) or wild-type (GSDMD I105N^+/+^) macrophages were treated with FlaTox in media containing DEVD probe and PI and imaged on an Incucyte platform. Percentages of all Incucyte experiments were calculated as the number of positive cells relative to a PI-stained, Triton-x100-treated well (considered 100%). Values represent mean ± SD of technical duplicates of a representative experiment from three biological repeats. All scale bars represent 10 μm.

Based on our detection of DEVDase activity in pyroptotic macrophages and the observation that caspase-1 and caspase-8 redundantly activate executioner caspase-3 and caspase-7, we hypothesized that GSDMD-induced pore formation and cell lysis may mask a background apoptotic program in pyroptotic cells. In order to eliminate confounding effects of GSDMD-mediated cell lysis, we further dissected the function and signaling mechanism of this caspase cascade in a GSDMD-deficient background. Consistent with previous reports showing that canonical inflammasome activation triggers an alternative cell death response in GSDMD-deficient macrophages (de Vasconcelos et al., 2019; Gonçalves et al., 2019; He et al., 2015; Kayagaki et al., 2015; Tsuchiya et al., 2019), release of the lytic cell death marker lactate dehydrogenase (LDH) was blunted in culture media of LeTx-treated B6^Nlrp1b+^*Gsdmd*^−/−^ cells and FlaTox-stimulated *Gsdmd*^−/−^ BMDMs (Figures 3A and 3B). However, careful analysis of DIC micrographs showed cells with a shrunken and blebbing appearance that are reminiscent of apoptosis and distinct from the classical swollen morphology of pyroptotic macrophages (Figures 3C and 3D). In agreement, flow cytometric analysis of LeTx- and FlaTox-induced cell death in respectively B6^Nlrp1b+^*Gsdmd*^−/−^ and *Gsdmd*^−/−^ BMDMs identified a population of ∼45% apoptotic cells that was positive for the early apoptosis marker Annexin-V while being impermeable to PI (Annexin-V^+^/PI^−^). In contrast, GSDMD-proficient pyroptotic macrophages displayed Annexin-V and PI co-staining (Annexin-V^+^/PI^+^) (Figures 3E and 3F). A kinetic analysis of DEVDase activity further corroborated these results. The number of DEVDase-positive cells was comparable in pyroptotic B6^Nlrp1b+^ and apoptotic B6^Nlrp1b+^*Gsdmd*^−/−^ macrophages following LeTx or FlaTox stimulation (Figure 3G). DEVDase activity was delayed in apoptotic macrophages relative to pyroptotic cells (Figures 3G and 3H), although this could at least partially be due to less efficient cytosolic uptake of the fluorogenic substrate in early apoptotic cells. Notably, BMDMs from mice expressing an inactive GSDMD^I105N^ mutant that is impaired in inducing pyroptotic cell lysis (Kayagaki et al., 2015) phenocopied *Gsdmd*^−/−^ macrophages (Figure 3I), demonstrating that inflammasome-mediated apoptosis induction was not unique to GSDMD-deficient macrophages and that impaired GSDMD pore formation downstream of caspase-1 and caspase-8 activation suffices to unmask inflammasome-induced apoptotic hallmarks in macrophages. Unlike B6^Nlrp1b+^*Gsdmd*^−/−^ macrophages, *Gsdmd*^−/−^ macrophages (that lack expression of a LeTx-responsive *Nlrp1b* allele) failed to induce apoptosis (Figures S2A and S2B) as well as PI staining and DEVDase activity (Figure S2C) in response to LeTx intoxication. Paralleling results in LeTx-intoxicated B6^Nlrp1b+^ macrophages (Figures 1B and S1D), the proteasome inhibitor MG132 inhibited the induction of DEVDase activity and PI staining in LeTx-stimulated B6^Nlrp1b+^*Gsdmd*^−/−^ macrophages (Figure S2D). Thus, expression of a functional *Nlrp1b* allele is required for LeTx-induced apoptosis in GSDMD-deficient macrophages. Similarly, we confirmed that Nlrc4 acts upstream of FlaTox-induced apoptosis, because *Nlrc4*^−/−^*Gsdmd*^−/−^ macrophages were unresponsive to FlaTox (Figure S2E**)**. These results demonstrate that activation of the Nlrp1b and Nlrc4 inflammasomes in the absence of GSDMD expression culminates in apoptosis.

Next, we compared the caspase cascade that underlies the apoptotic response in GSDMD-deficient BMDMs to the set of caspases that is activated during pyroptosis in GSDMD-proficient (wild-type) macrophages. Consistent with its apical role in inflammasome signaling, we observed prominent caspase-1 maturation in lysates of both apoptotic B6^Nlrp1b+^*Gsdmd*^−/−^ and pyroptotic B6^Nlrp1b+^ macrophages that had been stimulated with LeTx (Figure 3J). Similarly, FlaTox potently induced caspase-1 maturation in apoptotic *Gsdmd*^−/−^ and pyroptotic wild-type (B6) macrophages (Figure 3J). Consistent with previous reports (Gurung et al., 2014; Man et al., 2013; Van Opdenbosch et al., 2017), weak maturation of procaspase-8 was observed in pyroptotic macrophages (Figure 3J). LeTx- and FlaTox-induced apoptosis in GSDMD-deficient macrophages was associated with substantially increased caspase-8 cleavage (Figure 3J). Notably, levels of caspase-7 maturation were comparable in pyroptotic and apoptotic cells (Figure 3J), whereas caspase-3 cleavage was more prominent during apoptosis (Figure 3J). Nlrp1b signaling was required for LeTx-induced apoptotic caspase activation because activation of caspase-1, caspase-3, caspase-7, and caspase-8 was abolished in *Gsdmd*^−/−^ macrophages that lack a LeTx-responsive *Nlrp1b* allele (Figure S2F**)**. Together, these findings support the notion of an apoptotic caspase activation network in pyroptotic cells that is further accentuated in the absence of GSDMD-mediated cell lysis.

### Caspase-1 and Caspase-8 Independently Activate Caspase-3 and Caspase-7 and Apoptosis in GSDMD-Deficient Macrophages

**Figure 4:**
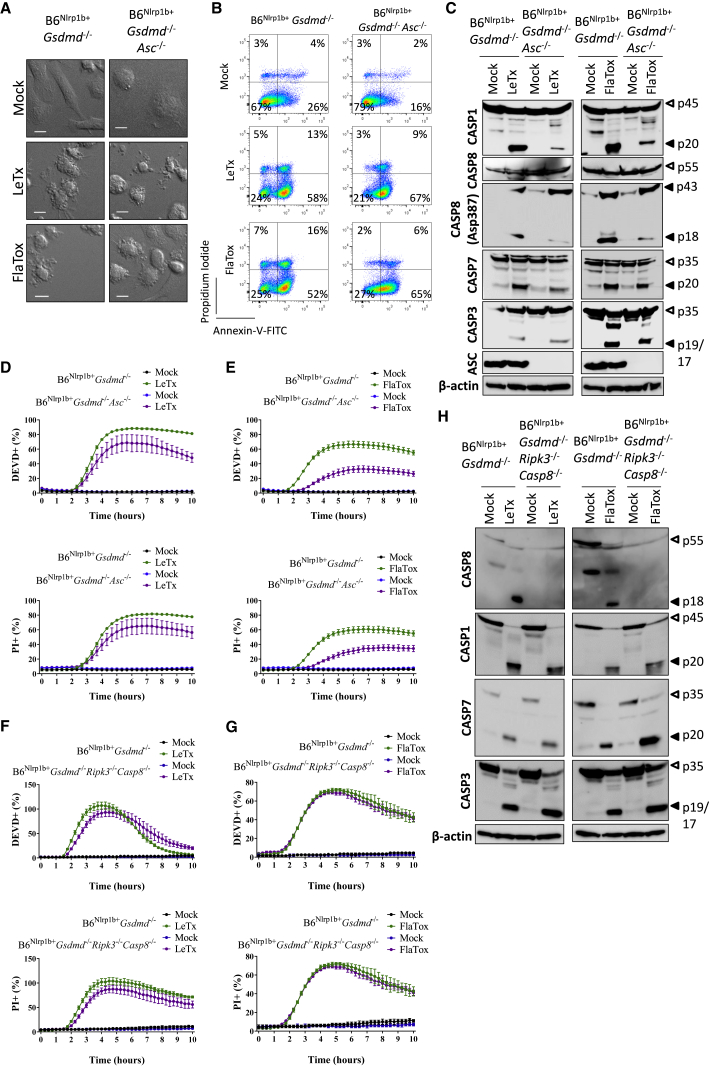
Caspase-1 and Caspase-8 Act Redundantly in GSDMD-Deficient Macrophages for Activation of Caspase-3 and Caspase-7 (A–C) Macrophages of the indicated genotypes were left untreated or stimulated with either LeTx or FlaTox for 2 h. Cells were imaged under a confocal microscope (A) or analyzed by FACS for Annexin-V/PI positivity (B), and cell lysates were immunoblotted for the indicated proteins (C). (D and E) Macrophages of the indicated genotypes were left untreated or stimulated with either LeTx (D) or FlaTox (E) in media containing DEVD probe and PI and imaged on an Incucyte platform. (F and G) Macrophages of the indicated genotypes were left untreated or stimulated with either LeTx (F) or FlaTox (G) in media containing DEVD probe and PI and imaged on an Incucyte platform. (H) Macrophages of the indicated genotypes were left untreated or stimulated with either LeTx or FlaTox for 2 h, and cell lysates were immunoblotted for the indicated proteins. Percentages of all Incucyte experiments were calculated as the number of positive cells relative to a PI-stained, Triton-x100-treated well (considered 100%). Values represent mean ± SD of technical duplicates of a representative experiment from three biological repeats. All scale bars represent 10 μm.

We and others (Lee et al., 2018; Mascarenhas et al., 2017; Van Opdenbosch et al., 2017) have shown that ASC is critical for LeTx- and FlaTox-induced caspase-8 activation and induction of apoptosis in caspase-1-deficient macrophages (Figure S1I). In marked contrast, ASC deletion failed to protect GSDMD-deficient BMDMs from undergoing LeTx- and FlaTox-induced apoptosis (Figures 4A and 4B). Furthermore, western blot analysis revealed that – albeit significantly reduced - B6^Nlrp1b+^*Gsdmd*^−/−^*Asc*^−/−^ macrophages continued to mature caspase-8 (Figure 4C). Moreover, cleavage of the apoptotic executioner caspase-3 and caspase-7 was abundant in both ASC-sufficient and ASC-deficient B6^Nlrp1b+^*Gsdmd*^−/−^ macrophages (Figure 4C). In agreement, LeTx- and FlaTox-induced DEVDase activity was reduced, but not abolished, in B6^Nlrp1b+^*Gsdmd*^−/−^*Asc*^−/−^ macrophages (Figures 4D and 4E). These results imply that activation of caspase-1 in GSDMD-deficient cells elicits maturation of caspase-8, caspase-3, and caspase-7 independently of the previously uncovered ASC-caspase-8 axis that drives apoptosis in caspase-1-deficient cells (Lee et al., 2018; Mascarenhas et al., 2017; Van Opdenbosch et al., 2017). Transgenic overexpression of anti-apoptotic Bcl2 in BMDMs of B6^Nlrp1b+^*Gsdmd*^−/−^*Bcl2*^*Tg*^ mice (Domen et al., 1998) failed to curb the kinetics of LeTx- or FlaTox-induced DEVDase activity (Figures S2G and S2H), suggesting that Bax/Bak pore formation may not be critical for inflammasome-mediated activation of apoptotic executioner caspases.

In order to directly assess the role of caspase-8, we generated *Ripk3*^−/−^*Casp8*^−/−^ macrophages in a B6^Nlrp1b+^*Gsdmd*^−/−^ background. A dynamic analysis of DEVDase activity and PI incorporation demonstrated that these cell death markers were comparably induced in caspase-8/Ripk3-deficient and control B6^Nlrp1b+^*Gsdmd*^−/−^ macrophages (Figures 4F and 4G). Additionally, LeTx- and FlaTox-induced maturation of caspase-1 and apoptotic executioner caspase-3 and caspase-7 in these cells was comparable to levels seen in caspase-8/Ripk3-sufficient B6^Nlrp1b+^*Gsdmd*^−/−^ BMDMs (Figure 4H). These findings demonstrate that in marked contrast to caspase-1-deficient macrophages (Lee et al., 2018; Van Opdenbosch et al., 2017), caspase-8 is dispensable for activation of caspase-3 and caspase-7 in macrophages lacking GSDMD expression. A functional implication of these results is that unlike in caspase-1-deficient macrophages (Lee et al., 2018; Van Opdenbosch et al., 2017), Toll-like receptor (TLR) priming in GSDMD-deficient macrophages fails to suppress LeTx- and FlaTox-stimulated maturation of caspase-1, caspase-8, caspase-3, and caspase-7 (Figure S3A); DEVDase activity (Figures S3B and S3C); and induction of apoptosis (Figures S3D and S3E).

### Nlrp3-Inflammasome-Induced Apoptosis in Gsdmd^−/−^ Macrophages

**Figure 5:**
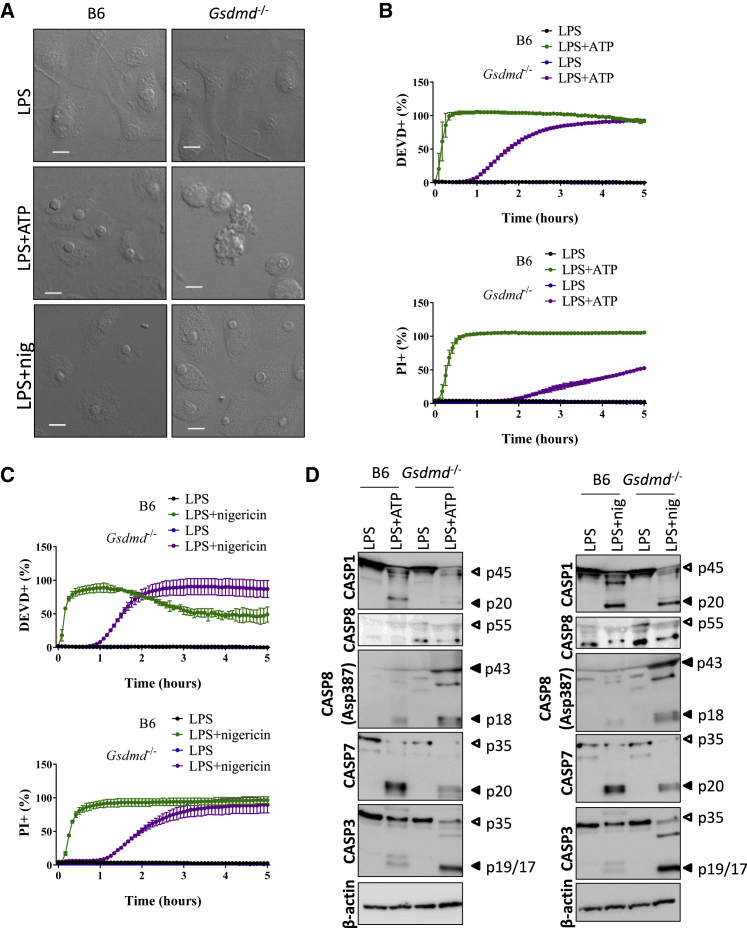
Nlrp3 Activation Promotes Apoptosis in GSDMD-Deficient Macrophages (A and D) Macrophages of the indicated genotypes were primed with LPS (100 ng/mL) for 3 h and left untreated or stimulated with ATP or nigericin (nig) for 2 h. Cells were imaged under a confocal microscope (A), and cell lysates were immunoblotted for the indicated proteins (D). (B and C) Macrophages of the indicated genotypes were primed with LPS (100 ng/mL) for 3 h, left untreated or stimulated with ATP (B) or nigericin (C) in DEVD and PI-containing media, and imaged on an Incucyte platform. Percentages of all Incucyte experiments were calculated as the number of positive cells relative to a PI-stained, Triton-x100-treated well (considered 100%). Values represent mean ± SD of technical duplicates of a representative experiment from three biological repeats. All scale bars represent 10 μm.

Lipopolysaccharide (LPS) priming is required for ATP- and nigericin-induced activation of the Nlrp3 inflammasome in BMDMs (Bauernfeind et al., 2009; Le Feuvre et al., 2002; Song et al., 2017). As expected, micrographs of LPS+ATP- and LPS+nigericin-stimulated wild-type macrophages displayed a characteristic pyroptotic morphology featuring a swollen cytosol and rounded nuclei (Figure 5A). As with pyroptosis induction by the Nlrp1b and Nlrc4 inflammasomes (Figure 1), Nlrp3-driven pyroptosis in LPS+ATP- and LPS+nigericin-stimulated wild-type macrophages was accompanied by a sharp rise in DEVDase activity concomitant with PI staining (Figures 5B and 5C). The morphology of ATP-stimulated *Gsdmd*^−/−^ macrophages differed considerably, with cytosolic shrinkage and formation of apoptotic bodies evident within minutes after ATP stimulation (Figure 5A). Furthermore, DEVDase activity in LPS+ATP-stimulated *Gsdmd*^−/−^ macrophages preceded the induction of PI staining by ∼1 h (Figure 5B), consistent with the induction of secondary necrosis upon prolonged *in vitro* incubation of apoptotic cells. Unexpectedly, LPS+nigericin-stimulated *Gsdmd*^−/−^ macrophages had a swollen appearance suggestive of necrotic cell death (Figure 5A), although a delayed induction of DEVDase activity that slightly preceded the induction of PI staining was observed (Figure 5C), suggesting that inflammasome-mediated apoptotic morphological changes may have been masked by osmotic disbalance directly mediated by the ionophore. In agreement, Nlrp3-mediated pyroptosis in wild-type BMDMs was associated with prominent maturation of caspase-1 and caspase-7, whereas *Gsdmd*^−/−^ macrophages additionally triggered robust cleavage of caspase-3 and caspase-8 following treatment with LPS+ATP or LPS+nigericin (Figure 5D). These results extend our observations on pyroptotic DEVDase activity to the Nlrp3 inflammasome and show that Nlrp3 activation in *Gsdmd*^−/−^ cells promotes induction of apoptotic cell death markers, akin to the Nlrp1b and Nlrc4 pathways.

### Caspase-3 and Caspase-7 Are Redundant for Apoptosis of GSDMD-Deficient Cells

**Figure 6:**
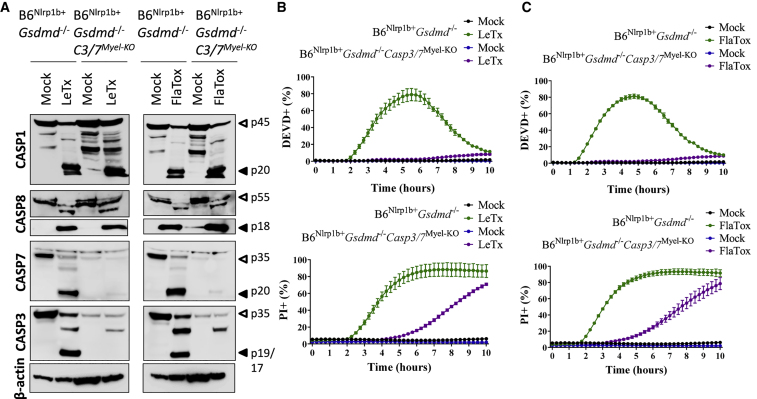
Caspase-3 and Caspase-7 Are Critical for Inflammasome-Induced Apoptosis of GSDMD-Deficient Macrophages (A) Immortalized macrophages of the indicated genotypes were left untreated or stimulated with either LeTx or FlaTox for 2 h, and cell lysates were immunoblotted for the indicated proteins. (B and C) Immortalized macrophages of the indicated genotypes were left untreated or stimulated with either LeTx (B) or FlaTox (C) in media containing DEVD probe and PI and imaged on an Incucyte platform. Percentages of all Incucyte experiments were calculated as the number of positive cells relative to a PI-stained, Triton-x100-treated well (considered 100%). Values represent mean ± SD of technical duplicates of a representative experiment from three biological repeats.

We next addressed the relative contributions of caspase-3 and caspase-7 to Nlrp1b- and Nlrc4- induced apoptosis signaling in *Gsdmd*^−/−^ macrophages. To this end, we differentiated BMDMs from B6^Nlrp1b+^*Gsdmd*^−/−^*Casp7*^*flox/flox*^ mice and induced *Casp7* gene deletion *in vitro* using cell-permeable active Cre protein (TAT-Cre) (Peitz et al., 2002). Caspase-7 protein levels were downregulated (Figure S4A); however, neither DEVDase activity nor PI incorporation was altered following LeTx or FlaTox stimulation (Figures S4B and S4C). Consistently, caspase-7 silencing had no effect on LeTx- and FlaTox-induced maturation of caspase-1, caspase-8, and caspase-3 (Figure S4A). Similarly, TAT-Cre-mediated excision of a floxed *Casp3* allele in B6^Nlrp1b+^*Gsdmd*^−/−^*Casp3*^*flox/flox*^ macrophages downregulated caspase-3 protein expression levels (Figure S4D). However, LeTx- and FlaTox-induced DEVDase activity and PI staining were unaffected (Figures S4E and S4F), and maturation of caspase-1, caspase-8, and caspase-7 were unchanged (Figure S4D). These results suggest that caspase-3 and caspase-7 are independently activated upon inflammasome activation and that the executioner caspases are jointly responsible for inducing DEVDase activity following inflammasome activation. To test this hypothesis, we generated B6^Nlrp1b+^*Gsdmd*^−/−^*Casp3*^*flox/flox*^*Casp7*^*flox/flox*^LysM-Cre^+^ (B6^Nlrp1b+^*Gsdmd*^−/−^*Casp3/7*^*Myel-KO*^) mice by breeding B6^Nlrp1b+^ mice with animals harboring conditionally targeted *Casp3* and *Casp7* alleles (*Casp3/7*^*F/F*^) (Saavedra et al., 2018) and subsequently to mice expressing Cre recombinase under control of the myeloid-cell-specific lysozyme M promoter (LysM-Cre) (Clausen et al., 1999). Immortalized bone marrow progenitor cells of these mice were used to generate estrogen-regulated homeobox protein Hox-B8 (ER-Hoxb8)-immortalized macrophages (iBMDMs) (Wang et al., 2006). As a reference, caspase-3/7-sufficient B6^Nlrp1b+^*Gsdmd*^−/−^ iBMDMs potently activated caspase-1, caspase-8, caspase-3, and caspase-7 following stimulation with LeTx or FlaTox (Figure 6A). Consistent with silencing of caspase-3 and caspase-7 protein expression levels in B6^Nlrp1b+^*Gsdmd*^−/−^*Casp3/7*^*Myel-KO*^ iBMDMs (Figure 6A), these cells failed to induce DEVDase activity in response to LeTx- and FlaTox stimulation up to 10 h post-stimulation (Figures 6B and 6C), confirming that DEVDase activity reflected the joint activation of caspase-3 and caspase-7 in inflammasome-stimulated *Gsdmd*^−/−^ macrophages. Notably, although plasma membrane permeabilization was delayed by several hours in the absence of caspase-3 and caspase-7, B6^Nlrp1b+^*Gsdmd*^−/−^*Casp3/7*^*Myel-KO*^ iBMDMs started to display prominent PI incorporation ∼6 h after stimulation with FlaTox or LeTx and approximated the levels of PI staining of caspase-3/7-sufficient cells by 10 h post-stimulation (Figure 6B and 6C). Because delayed cell lysis was not observed in cells lacking caspase-1 and caspase-8 (Figure S1) and activation of caspase-1 and caspase-8 was unaffected in B6^Nlrp1b+^*Gsdmd*^−/−^*Casp3/7*^*Myel-KO*^ iBMDMs (Figure 6A), the delayed lytic activity in B6^Nlrp1b+^*Gsdmd*^−/−^*Casp3/7*^*Myel-KO*^ iBMDMs may represent a yet-undefined cell death mechanism that is induced by these initiator caspases when GSDMD-mediated pore formation and caspase-3/7-driven apoptosis have failed. Alternatively, residual caspase-3/-7 activation levels in our system may account for the delayed cytotoxicity.

## Discussion

Macrophages that lack caspase-1 or express the catalytically inactive caspase-1^C284A^ mutant switch to caspase-8-mediated apoptosis (Pierini et al., 2012; Sagulenko et al., 2013; Van Opdenbosch et al., 2017). GSDMD-deficient macrophages were also suggested to switch cell death modes. *Legionella pneumophila* infection was reported to trigger caspase-7-mediated pore formation in GSDMD-deficient macrophages (Gonçalves et al., 2019), and other inflammasome stimuli were shown to induce caspase-3-mediated apoptosis and deafness associated tumor suppressor/gasdermin E (DFNA5/GSDME)-mediated secondary necrosis downstream of caspase-1 (Tsuchiya et al., 2019), or to switch to caspase-1-mediated cleavage of caspase-3 and caspase-7 and apoptotic cell death (Mahib et al., 2020; Taabazuing et al., 2017). However, the mechanisms by which inflammasomes regulate the switch from pyroptosis to apoptosis signaling remain unclear. Rather than switching cell death modes, our observations strongly suggest that an apoptotic program is readily activated concomitant with induction of GSDMD pores in wild-type macrophages and not only in the context of genetic deletion of caspase-1 or GSDMD. The presented findings give rise to a mechanistic model of pyroptosis in which caspase-1 cleaves GSDMD for cell lysis in parallel to caspase-1 and caspase-8 redundantly activating caspase-3 and caspase-7, both of which promote apoptotic DEVDase activity independently of each other in pyroptotic cells (Figure S5). In support of this model, we showed that impaired GSDMD pore formation downstream of caspase-1 and caspase-8 activation by the Nlrp1b and Nlrc4 inflammasomes sufficed to unveil apoptotic morphological features in GSDMD^I105N^ mutant macrophages. This novel paradigm of pyroptosis, in which activation of the apoptotic machinery is an intrinsic component of pyroptotic cell death signaling (Figure S5), has the merit that it elegantly explains how inflammasome-induced apoptotic hallmarks ensue through distinct signaling pathways in macrophages lacking caspase-1 or GSDMD, respectively.

In addition to the previously reported roles of ASC-mediated caspase-8 activation (Gonçalves et al., 2019; Pierini et al., 2012; Sagulenko et al., 2013; Van Opdenbosch et al., 2017), we now provided genetic evidence that also caspase-1 plays a critical role in inflammasome-induced apoptosis signaling. Indeed, combined deletion of caspase-1 and caspase-8 (or ASC in lieu of caspase-8) proved essential to blunt DEVDase activity and apoptosis induction in GSDMD-deficient cells. Moreover, we posit that executioner caspase-3 and caspase-7 act redundantly for inflammasome-induced apoptosis signaling, because combined deletion of caspase-3 and caspase-7 was necessary to blunt inflammasome-induced DEVDase activity and induction of cell death in GSDMD-sufficient and GSDMD-deficient macrophages, respectively. The redundancy we have uncovered between caspase-1 and caspase-8 as initiator caspases; and between GSDMD, and caspase-3 and caspase-7 in the execution phase of pyroptosis likely serves to ensure a commitment to cell death induction in inflammasome-activated macrophages. This built-in redundancy in the pyroptotic caspase cascade may have evolved to ensure the robustness of pyroptosis as an anti-microbial host defense mechanism. GSDMD-targeting pathogens remain to be discovered, but cowpox viruses express a cytokine response modifier (CrmA) that efficiently targets caspase-1 and caspase-8 (Zhou et al., 1997). This may have been an evolutionary more effective strategy for cowpox viruses to curb inflammasome-induced cell death than selective inhibition of caspase-1 or caspase-8. Moreover, the relative expression levels of caspase-1 and GSDMD, as well as other factors that regulate the kinetics of caspase-1-mediated GSDMD pore formation, may alter the balance of this integrated pyroptotic cell death program in favor of apoptosis as the default morphological outcome in non-myeloid cell types. For instance, it has been suggested that inflammasome-induced apoptosis may be the default inflammasome cell death mode in cell types that express no or low levels of GSDMD, such as primary cortical neurons, mast cells, keratinocytes, and endothelial cells (Sollberger et al., 2015; Tsuchiya et al., 2019; Xi et al., 2016). Although our studies have focused on macrophages (which express abundant levels of GSDMD), they suggest a mechanistic model of inflammasome-induced apoptosis that also may operate in cell types with low GSDMD levels. Finally, our results predict and clarify the mechanisms by which selective pharmacological GSDMD inhibitors will convert pyroptotic cell lysis into an apoptosis response that may curb detrimental inflammatory cytokine secretion in infectious and autoinflammatory diseases (de Vasconcelos and Lamkanfi, 2020; Kanneganti et al., 2018; Van Opdenbosch and Lamkanfi, 2019).

In conclusion, the presented work transforms understanding of pyroptosis from a linear signaling axis into an integrated cell death signaling network (Figure S5). Future studies should address whether caspase-3/7-mediated substrate cleavage in pyroptotic cells contributes to the quality of the instigated inflammatory and immune responses and how they impact on the resolution of infections.

## STAR★Methods

### Key Resources Table

**Table:** 

REAGENT or RESOURCE	SOURCE	IDENTIFIER
**Antibodies**		

Mouse monoclonal anti-caspase-1	Adipogen	Cat# AG-20B-0042-C100; RRID: AB_2755041; clone Casper-1
Mouse monoclonal anti-caspase-8	Enzo Life Sciences	Cat# ALX-804-447-C100; RRID: AB_2050952; clone 1G12
Rabbit monoclonal anti-cleaved caspase-8 (Asp387)	Cell Signaling	Cat# 8592S; RRID: AB_10891784; clone D5B2
Rabbit polyclonal anti-caspase-3	Cell Signaling	Cat# 9662; RRID: AB_331439
Rabbit monoclonal anti-cleaved caspase-3 (Asp175)	Cell Signaling	Cat# 9664S; RRID: AB_2070042; clone 5A1E
Rabbit polyclonal anti-caspase-7	Cell Signaling	Cat# 9492; RRID: AB_2228313
Rabbit polyclonal anti-cleaved caspase-7 (Asp198)	Cell Signaling	Cat# 9491; RRID: AB_2068144
Rabbit polyclonal anti-ASC	Adipogen	Cat# AG-25B-0006; RRID: AB_2490440
Rabbit monoclonal anti-GAPDH	Cell Signaling	Cat# 14C10; RRID: AB_561053; clone 2118
Rabbit monoclonal anti-ROCKI	Abcam	Cat# ab45171; RRID: AB_2182005; clone EP786Y
Goat polyclonal anti-Bid	R&D systems	Cat# AF860; RRID: AB_2065622
Goat polyclonal anti-mouse, HRP-conjugated	Jackson Immunoresearch Laboratories	Cat# 115-035-146; RRID: AB_2307392
Goat polyclonal anti–rabbit, HRP-conjugated	Jackson Immunoresearch Laboratories	Cat# 111-035-144; RRID: AB_2307391
Mouse monoclonal anti-β-actin, HRP-conjugated	US Biological	Cat# 137402.100

**Bacterial and Virus Strains**		

*Salmonella enterica* serovar Thypimurium, SL1344	In house	N/A

**Chemicals, Peptides, and Recombinant Proteins**		

LFn-FlaA	In house	(von Moltke et al., 2012)
B. anthracis protective antigen (PA)	In house	(von Moltke et al., 2012)
B. anthracis lethal factor (LF)	List Biologicals	Cat# 172C
TAT-Cre	In house	(Peitz et al., 2002)
LPS-SM	Invivogen	Cat# tlrl-smlps
MG132	Calbiochem	Cat# 474791
polymyxin B sulfate	Calbiochem	Cat# 5291
staurosporine	Selleckchem	Cat# S1421
cycloheximide	Sigma-Aldrich	Cat# C4859
leupeptin	Sigma-Aldrich	Cat# L8511
chloroquine	Sigma-Aldrich	Cat# C6628
β-estradiol	Sigma-Aldrich	Cat# E2758
ATP	Sigma-Aldrich	Cat# ATPD-RO
Enhanced chemiluminescence solution	Thermo Scientific	Cat# 32106
propidium iodide	Thermo Scientific	Cat# P1304MP
Sytox Green	Thermo Scientific	Cat# S7020
DEVD-based substrate	Thermo Scientific	Cat# R37111

**Critical Commercial Assays**		

CytoTox 96 non-radioactive cytotoxicity assay	Promega	Cat# G1780
annexin V-FITC and PI	BD PharMingen	Cat# 556547

**Experimental Models: Cell Lines**		

ER-Hoxb8 immortalized myeloid progenitor cells	In house	(Wang et al., 2006)

**Experimental Models: Organisms/Strains**		

Mouse: B6: C57BL/6J	In house	Charles River
Mouse: B6^Nlrp1b+^: C57BL/6J^Nlrp1b+Tg/WT^	In house	(Boyden and Dietrich, 2006)
Mouse: *Casp1*^−/−^/*Casp11*^−/−^	In house	(Kuida et al., 1995)
Mouse: *Nlrc4*^−/−^		(Mariathasan et al., 2004)
Mouse: Bcl2^Tg^: C57BL/6J^H2K-Bcl2Tg/WT^	In house	(Domen et al., 1998)
Mouse: *Gsdmd*^−/−^	In house	(Kayagaki et al., 2015)
Mouse: GSDMD^I105N^: C57BL/6J - GSDMD ^I105N/I105N^	In house	(Kayagaki et al., 2015)
Mouse: *Ripk3*^−/−^*Casp8*^−/−^	In house	(Newton et al., 2014)
Mouse: *Asc*^−/−^	In house	(Mariathasan et al., 2004)
Mouse: *Casp3/7*^*flox/flox*^: C57BL/6J - *Casp3*^*flox/flox*^/*Casp7*^*flox/flox*^	In house	(Saavedra et al., 2018)
Mouse: *Casp3/7*^*Myel-KO*^: C57BL/6J - *Casp3*^*flox/flox*^*Casp7*^*flox/flox*^LysM-Cre^+^	In house	(Clausen et al., 1999; Saavedra et al., 2018)

**Software and Algorithms**		

Fiji	NIH	N/A
FlowJo	FlowJo LLC	N/A
Prism 8	GraphPad	N/A
Incucyte Zoom	Essenbio	N/A

### Resource Availability

#### Lead Contact

Further information and requests for resources and reagents should be directed to and will be fulfilled by the Lead Contact, Mohamed Lamkanfi (mohamed.lamkanfi@ugent.be).

#### Materials Availability

This study did not generate new unique reagents.

#### Data and Code Availability

This study did not generate unique datasets or codes.

### Experimental Model and Subject Details

#### Mice

B6^Nlrp1b+^ (Boyden and Dietrich, 2006), *Gsdmd*^−/−^ (Kayagaki et al., 2015), GSDMD^I105N^ (Kayagaki et al., 2015), *Ripk3*^−/−^*Casp8*^−/−^ (Newton et al., 2014), *Casp1*^−/−^/*Casp11*^−/−^ (Kuida et al., 1995), H2K-Bcl2^Tg^ (Bcl2^Tg^) (Domen et al., 1998), *Nlrc4*^−/−^ and *Asc*^−/−^ (Mariathasan et al., 2004), Lysozyme M (Clausen et al., 1999) and *Casp3*^*flox/flox*^/*Casp7*^*flox/flox*^ (Saavedra et al., 2018) mice have been described before. Animals were housed in individually ventilated cages under specific pathogen-free conditions, and males and females were used between the age of 8 and 12 weeks. Studies were conducted under protocols approved by Ghent University Committee on Use and Care of Animals.

### Method Details

#### Primary macrophage differentiation

Macrophages were differentiated by culturing bone marrow progenitor cells in Iscove’s modified Dulbecco’s medium (IMDM; Lonza) containing 10% (v/v) heat-inactivated FBS, 30% (v/v) L929 cell-conditioned medium, 1% (v/v) non-essential amino acids (Lonza), 100 U/ml penicillin and 100 mg/ml streptomycin at 37°C in a humidified atmosphere containing 5% CO_2_ for six days. Bone marrow-derived macrophages (BMDMs) were then seeded into 96 or 12 well plates as needed, in IMDM containing 10% FBS, 1% non-essential amino acids and antibiotics.

#### Immortalized macrophage generation and differentiation

Generation of ER-Hoxb8 immortalized myeloid progenitor cells was performed as previously described (Wang et al., 2006). Briefly, bone marrow progenitor cells were infected with ER-Hoxb8 retrovirus. Cells were selected with RPMI 1640 medium (Lonza) containing 10% (v/v) heat-inactivated FBS, 100 U/ml penicillin and 100 mg/ml streptomycin, 1% (v/v) B16 cell-conditioned medium and β-estradiol (1 μM) at 37°C in a humidified atmosphere containing 5% CO_2_. Cell cultures were passaged every two to three days into new media containing fresh β-estradiol and B16 cell-conditioned medium. For differentiation into immortalized bone marrow-derived macrophages (iBMDM), progenitor cells were counted and washed once with PBS. Then, the same protocol for primary BMDMs differentiation was followed.

#### Macrophage stimulation

The next day after seeding primary or immortalized macrophages, cells were changed to fresh media and either left untreated or stimulated with log phase *S.* Typhimurium (MOI 5); TNFα (20 ng/ml) and cycloheximide (50 μg/ml); anthrax protective antigen (PA, 1 μg/ml) plus either lethal factor (500 ng/ml) or LFn-FlaA (1 μg/ml); staurosporine (1 μM). Alternatively, BMDMs were primed with LPS (100 ng/ml) for 3h prior to stimulation with LeTx, FlaTox, nigericin (20 μM) or ATP (5 mM) or treated with the proteasome inhibitor MG132 (10 μM) for 30 min prior to LeTx incubation.

#### TAT-Cre-mediated deletion of floxed alleles

In order to specifically delete floxed alleles, B6^Nlrp1b+^*Gsdmd*^−/−^*Casp3*^*flox/flox*^, B6^Nlrp1b+^*Gsdmd*^−/−^*Casp7*^*flox/flox*^ or B6^Nlrp1b+^*Casp3/7*^*flox/flox*^ macrophages were collected at day 6 post differentiation and seeded into Petri dishes. For two consecutive days, cells had their media changed to 5 mL IMDM containing TAT-Cre (1,5 μM), chloroquine (200 μM), leupeptin (2 μM) and polymyxin B (60 μg/ml) for 1 h at 37°C and then washed to fresh media. As controls, either B6^Nlrp1b+^*Gsdmd*^−/−^ macrophages were treated in parallel with the same TAT-Cre-containing mix or B6^Nlrp1b+^*Casp3/7*^*flox/flox*^ cells received the treatment in the absence of TAT-Cre. After two days, cells were scraped, counted and seeded in 96 or 12 well plates, as needed.

#### Western blotting

Cells were lysed with lysis buffer (20 mM Tris HCl pH 7.4, 200 mM NaCl, 1% NP-40) and denatured in Laemmli buffer. For detection of caspase-1, ASC, caspase-3, caspase-7, ROCKI and Bid, a part of the supernatant was kept with the cell lysates. For detection of caspase-8, most of the supernatant was removed. Protein samples were boiled at 95°C for 10 min before being separated by SDS-PAGE and transferred to polyvinylidene difluoride (PVDF) membranes. Blocking, incubation with antibody and washing of the membrane were done in PBS supplemented with 0.05% or 0.2% Tween-20 (v/v) and 3% non-fat dry milk. Immunoblots were incubated overnight with primary antibodies. Secondary antibodies were used to detect proteins by enhanced chemiluminescence.

#### Cell death kinetic measurements

A plate-based fluorescent assay was used to quantify cell permeabilization based on propidium iodide (PI, 0,1 μg/ml) or Sytox Green (SG, 5μM) incorporation and fluorescence from a DEVD-based substrate, used according to the manufacturers’ instructions. Data were acquired and analyzed to obtain the fluorescent object count using the Incucyte Zoom system (Essenbio). Briefly, cells plated on a 96 well plate were pre-incubated with the reagents for 2h, stimulated and incubated in a CO_2_ and temperature-controlled environment that allowed measurement of fluorescent signals over a time span of 10 hr. Values were normalized against a control well that was seeded with the same number of cells of the same genotype as in the experimental well and imaged in parallel following treatment with Triton-x-100 to fully permeabilize the cells. The maximum number of positive nuclei in these control wells was considered 100% and used to normalize values of fluorescent cells in experimental wells.

#### FACS Annexin-V measurements

Annexin-V and PI staining on cells was performed according to the manufacturer’s instructions. Flow cytometry was used to measure stained cells and data were analyzed with FlowJo Software.

#### Confocal imaging

BMDMs plated on μ-slide wells (ibidi) were treated and imaged using an observer Z.1 spinning disk microscope (Zeiss, Zaventem, Belgium) equipped with a Yokogawa disk CSU-X1. Cells were incubated in a chamber with a 5% CO_2_ atmosphere at 37°C throughout the imaging experiment. DIC images were acquired with the use of a pln Apo 40x/1.4 oil DIC III objective and a Rolera em-c2 camera. Representative images were extracted and edited in Fiji (NIH).

#### LDH release

Supernatants of stimulated BMDMs were collected and centrifuged at 300xg for 5min to remove cellular debris. LDH measurement was performed with the CytoTox 96 Non-Radioactive Cytotoxicity Assay kit according to the manufacturers’ instructions, in samples diluted 1:5 in PBS. Data were plotted considering the O.D. value obtained from a well treated with Triton-x100 as 100% for each genotype.

### Quantification and Statistical Analysis

Statistical parameters including the definition of center, dispersion, and precision measures (mean ± SD) are reported in the figures and figure legends. All error bars represent mean values ± standard deviations of technical replicates obtained from a representative experiment out of three independent biological repeats. GraphPad Prism 8 (GraphPad Inc.) was used for analyzing and plotting data.

## Supplemental Information

Document S1. Figures S1–S5

Document S2. Article plus Supplemental Information
