# Supplementary material for: An Apoptotic Caspase Network Safeguards Cell Death Induction in Pyroptotic Macrophages
Source: Cell Rep. 2020 Jul 28;32(4):107959. doi: 10.1016/j.celrep.2020.107959 (PMC7408007; doi:10.1016/j.celrep.2020.107959)
Supplement: Document S1. Figures S1–S5 [file mmc1.pdf]

Cell Reports, Volume *32*

Supplemental Information

An Apoptotic Caspase Network Safeguards

Cell Death Induction in Pyroptotic Macrophages

Nathalia Moraes de Vasconcelos, Nina Van Opdenbosch, Hanne Van Gorp, Rosa Martín-Pérez, Annalisa Zecchin, Peter Vandenabeele, and Mohamed Lamkanfi

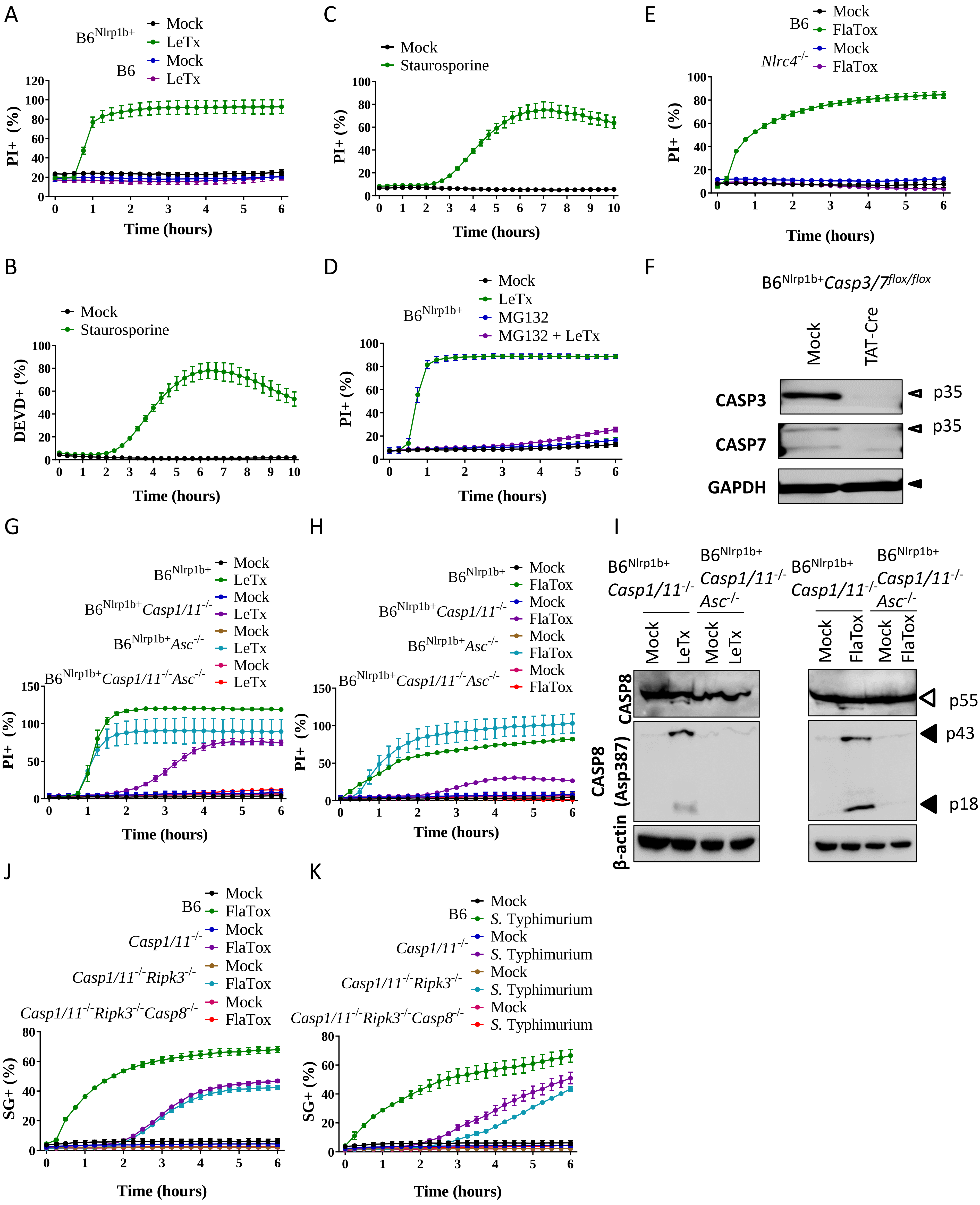
A
B6Nlrp1b+ Mock
LeTx
B6 Mock
LeTx
PI+ (%)
Time (hours)
B
Mock
Staurosporine
DEVD+ (%)
Time (hours)
C
Mock
Staurosporine
PI+ (%)
Time (hours)
D
B6Nlrp1b+ Mock
LeTx
MG132
MG132 + LeTx
PI+ (%)
Time (hours)
E
B6 Mock
FlaTox
Nlrc4-/- Mock
FlaTox
PI+ (%)
Time (hours)
F
B6Nlrp1b+Casp3/7flox/flox
Mock
TAT-Cre
CASP3
CASP7
GAPDH
p35
p35
G
B6Nlrp1b+ Mock
LeTx
B6Nlrp1b+Casp1/11-/- Mock
LeTx
B6Nlrp1b+Asc-/- Mock
LeTx
B6Nlrp1b+Casp1/11-/-Asc-/- Mock
LeTx
PI+ (%)
Time (hours)
H
B6Nlrp1b+ Mock
FlaTox
B6Nlrp1b+Casp1/11-/- Mock
FlaTox
B6Nlrp1b+Asc-/- Mock
FlaTox
B6Nlrp1b+Casp1/11-/-Asc-/- Mock
FlaTox
PI+ (%)
Time (hours)
I
B6Nlrp1b+ Casp1/11-/-
B6Nlrp1b+ Casp1/11-/- Asc-/-
Mock
LeTx
Mock
LeTx
Mock
FlaTox
Mock
FlaTox
CASP8
CASP8 (Asp387)
β-actin
p55
p43
p18
J
B6 Mock
FlaTox
Casp1/11-/- Mock
FlaTox
Casp1/11-/-Ripk3-/- Mock
FlaTox
Casp1/11-/-Ripk3-/-Casp8-/- Mock
FlaTox
SG+ (%)
Time (hours)
K
B6 Mock
S. Typhimurium
Casp1/11-/- Mock
S. Typhimurium
Casp1/11-/-Ripk3-/- Mock
S. Typhimurium
Casp1/11-/-Ripk3-/-Casp8-/- Mock
S. Typhimurium
SG+ (%)
Time (hours)

Fig. S1

Figure S1. Nlrp1b and Nlrc4-induced pyroptosis features a caspase-3/7 signature, related to Figure 1 and 2. (A, E, G, H, J, K) Macrophages of the indicated genotypes were left untreated or stimulated with LeTx, FlaTox or log phase *S.* Typhimurium in media containing PI or SG and imaged on an Incucyte platform. (B, C) Wildtype macrophages were left untreated or stimulated with staurosporine in media containing the DEVD-probe (B) or PI (C) and imaged on an Incucyte platform. (D) Macrophages of the indicated genotype either received MG132 (10μM) or not for 30 minutes and were left untreated or stimulated with LeTx in media containing the PI and imaged on an Incucyte platform. (F) Macrophages of the indicated genotype were left untreated or received TAT-Cre (as described in STAR Methods), and cell lysates immunoblotted for the indicated proteins. (I) Macrophages of the indicated genotypes were left untreated or stimulated with LeTx or Flatox for 2h, and cell lysates immunoblotted for the indicated proteins. Percentages of all Incucyte experiments were calculated as the number of positive cells relative to a PI or SG stained, Triton-x100-treated well (considered 100%). Values represent mean ± SD of technical duplicates of a representative experiment from three biological repeats.

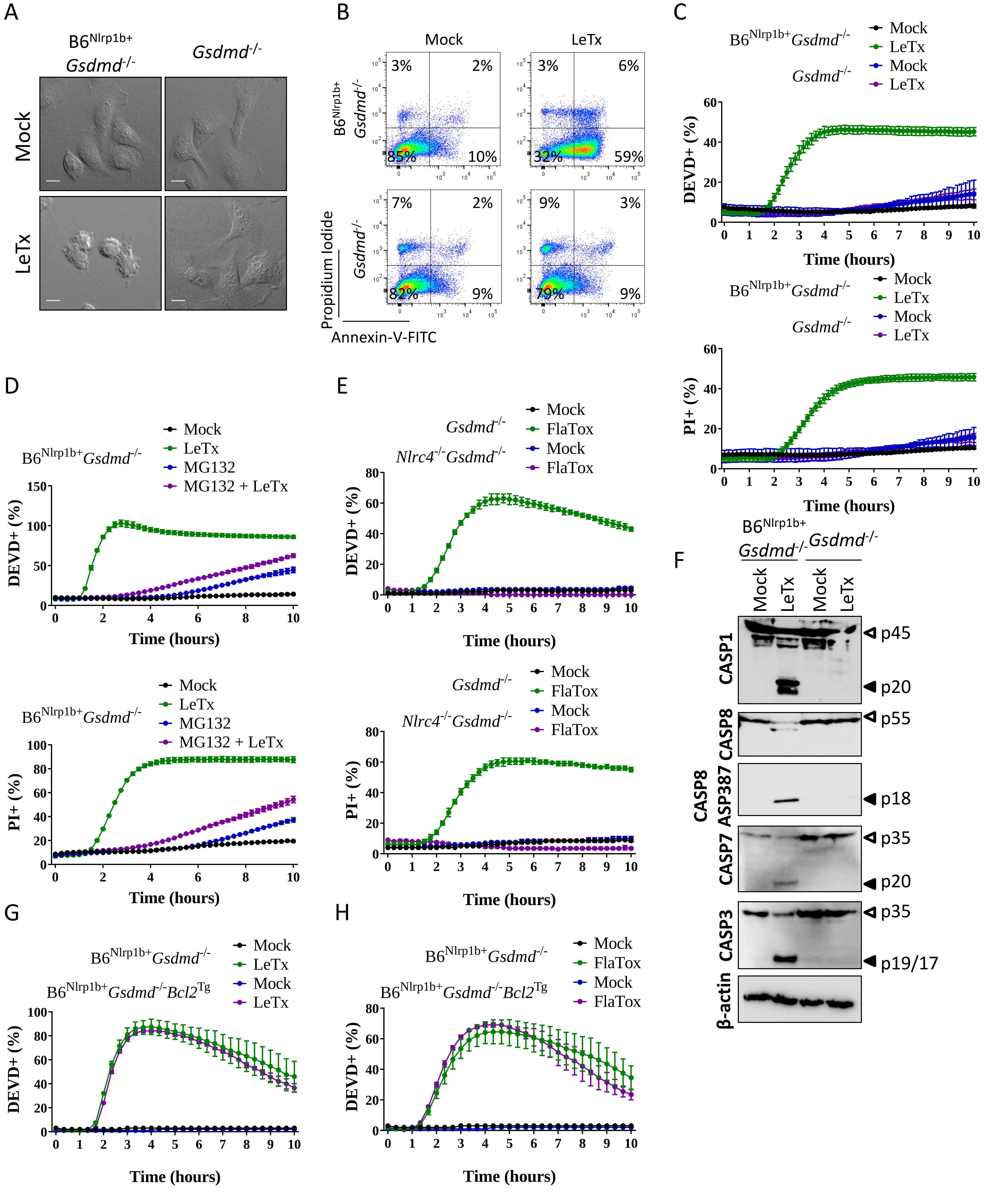
A
B6Nlrp1b+ Gsdmd-/-
Gsdmd-/-
Mock
LeTx
B
Mock
LeTx
Propidium Iodide
Annexin-V-FITC
C
DEVD+ (%)
PI+ (%)
Time (hours)
D
MG132
MG132 + LeTx
E
Nlrc4-/-Gsdmd-/-
FlaTox
F
CASP1
CASP8
CASP8 ASP387
CASP7
CASP3
β-actin
p45
p20
p55
p18
p35
p19/17
G
B6Nlrp1b+Gsdmd-/-Bcl2Tg
H

Fig. S2

Figure S2. Nlrp1b and Nlrc4 mediate LeTx- and FlaTox-induced apoptosis in GSDMD-deficient macrophages, related to Figure 3. (A, B) Macrophages of the indicated genotypes were left untreated or stimulated with LeTx for 2h, and cells were imaged under a confocal microscope (A) or analyzed by FACS for Annexin V/PI positivity (B). (C, E, G, H) Macrophages of the indicated genotypes were left untreated or stimulated with LeTx or FlaTox in media containing DEVD-probe or PI and imaged on an Incucyte platform. (D) Macrophages of the indicated genotype either received MG132 (10µM) or not for 30 minutes and were left untreated or stimulated with LeTx in media containing the DEVD-probe and PI and imaged on an Incucyte platform. (F) Macrophages of the indicated genotypes were left untreated or stimulated with LeTx for 2h and cell lysates immunoblotted for the indicated proteins. Percentages of all Incucyte experiments were calculated as the number of positive cells relative to a PI stained, Triton-x100-treated well (considered 100%). Values represent mean ± SD of technical duplicates of a representative experiment from three biological repeats. All scale bars, 10 µm.

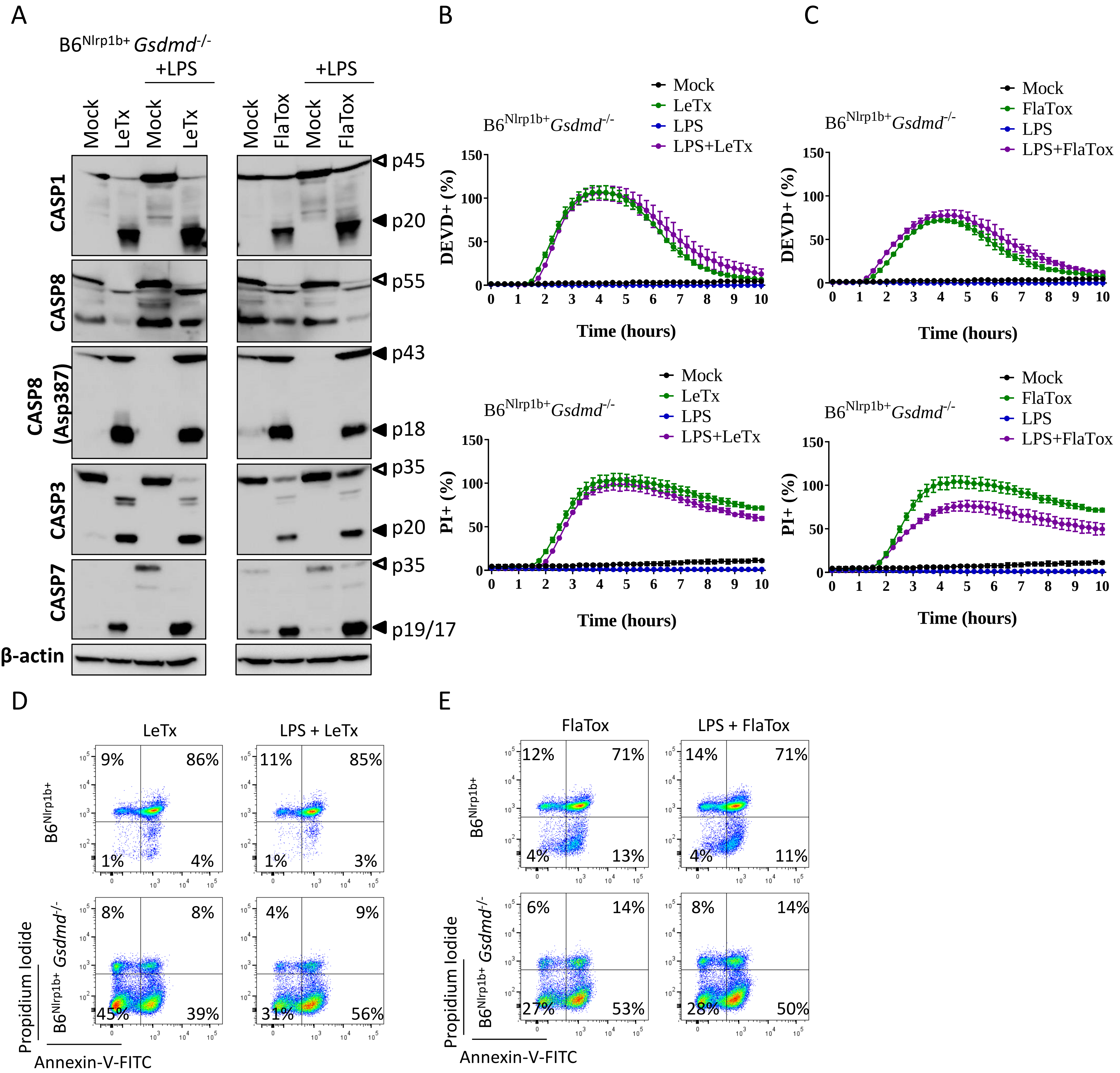
A
B6Nlrp1b+ Gsdmd-/-
+LPS
+LPS
Mock
LeTx
Mock
LeTx
Mock
FlaTox
Mock
FlaTox
CASP1
CASP8
CASP8 (Asp387)
CASP3
CASP7
β-actin
p45
p20
p55
p43
p18
p35
p20
p35
p19/17
B
C
B6Nlrp1b+Gsdmd-/-
Mock
LeTx
LPS
LPS+LeTx
Mock
FlaTox
LPS
LPS+FlaTox
DEVD+ (%)
PI+ (%)
Time (hours)
D
E
LeTx
LPS + LeTx
FlaTox
LPS + FlaTox
B6Nlrp1b+
B6Nlrp1b+ Gsdmd-/-
Propidium Iodide
Annexin-V-FITC
9%
86%
1%
4%
11%
85%
1%
3%
8%
8%
45%
39%
4%
9%
31%
56%
12%
71%
4%
13%
14%
71%
4%
11%
6%
14%
27%
53%
8%
14%
28%
50%

Fig. S3

Figure S3. Inflammasome-induced apoptosis in GSDMD-deficient macrophages is insensitive to suppression by LPS priming, related to Figure 4. (A, D, E) Macrophages of the indicated genotypes were primed or not with LPS (100ng/ml) for 3h and left untreated or stimulated with LeTx or FlaTox for 2h and cell lysates immunoblotted for the indicated proteins (A) or cells analyzed by FACS for Annexin V/PI positivity (D, E). (B, C) Macrophages of the indicated genotypes were primed or not with LPS (100ng/ml) for 3h and left untreated or stimulated with LeTx (B) or FlaTox (C) in DEVD and PI-containing media and imaged on a Incucyte platform. Percentages of all Incucyte experiments were calculated as the number of positive cells relative to a PI stained, Triton-x100-treated well (considered 100%). Values represent mean ± SD of technical duplicates of a representative experiment from three biological repeats.

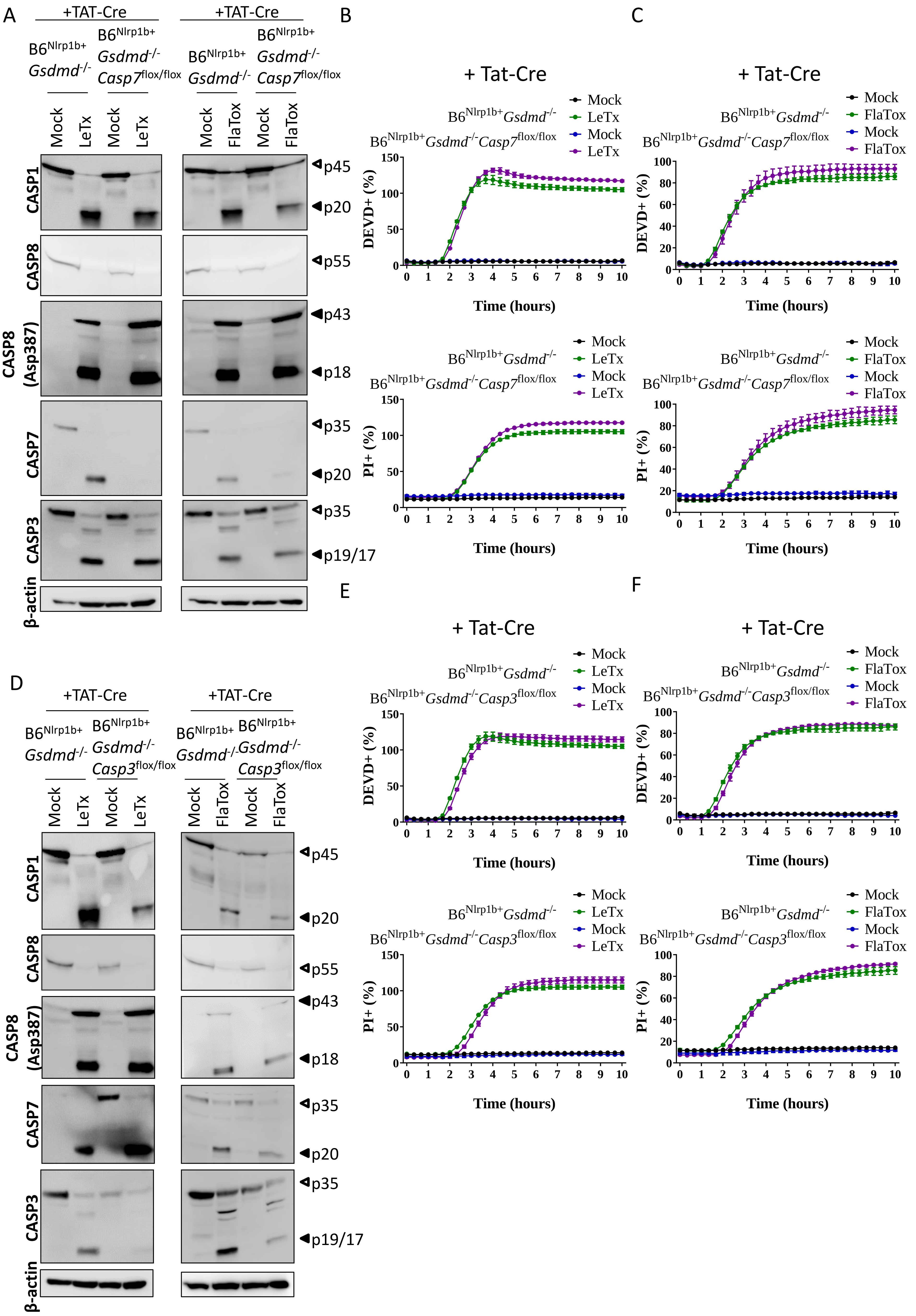
A
+TAT-Cre
B6Nlrp1b+ Gsdmd-/-
B6Nlrp1b+ Gsdmd-/- Casp7flox/flox
Mock LeTx Mock LeTx
Mock FlaTox Mock FlaTox
CASP1
CASP8
CASP8 (Asp387)
CASP7
CASP3
β-actin
p45
p20
p55
p43
p18
p35
p19/17
B
C
+ Tat-Cre
DEVD+ (%)
PI+ (%)
Time (hours)
Mock
LeTx
FlaTox
B6Nlrp1b+Gsdmd-/-
B6Nlrp1b+Gsdmd-/-Casp7flox/flox
D
B6Nlrp1b+ Gsdmd-/- Casp3flox/flox
E
F
B6Nlrp1b+Gsdmd-/-Casp3flox/flox

Fig. S4

Figure S4. Single deletion of caspase-3 or 7 fails to suppress inflammasome-induced apoptosis in GSDMD-deficient macrophages, related to Figure 6. (A, D) Macrophages of the indicated genotypes received TAT-Cre as described in *Methods* and were left untreated or stimulated with LeTx or FlaTox for 2h and cell lysates immunoblotted for the indicated proteins. (B, C, E, F) Macrophages of the indicated genotypes received TAT-Cre (as described in STAR Methods), and were left untreated or stimulated with LeTx (B and E) or FlaTox (C and F) in DEVD and PI-containing media and imaged on a Incucyte platform. Percentages of all Incucyte experiments were calculated as the number of positive cells relative to a PI stained, Triton-x100-treated well (considered 100%). Values represent mean ± SD of technical duplicates of a representative experiment from three biological repeats.

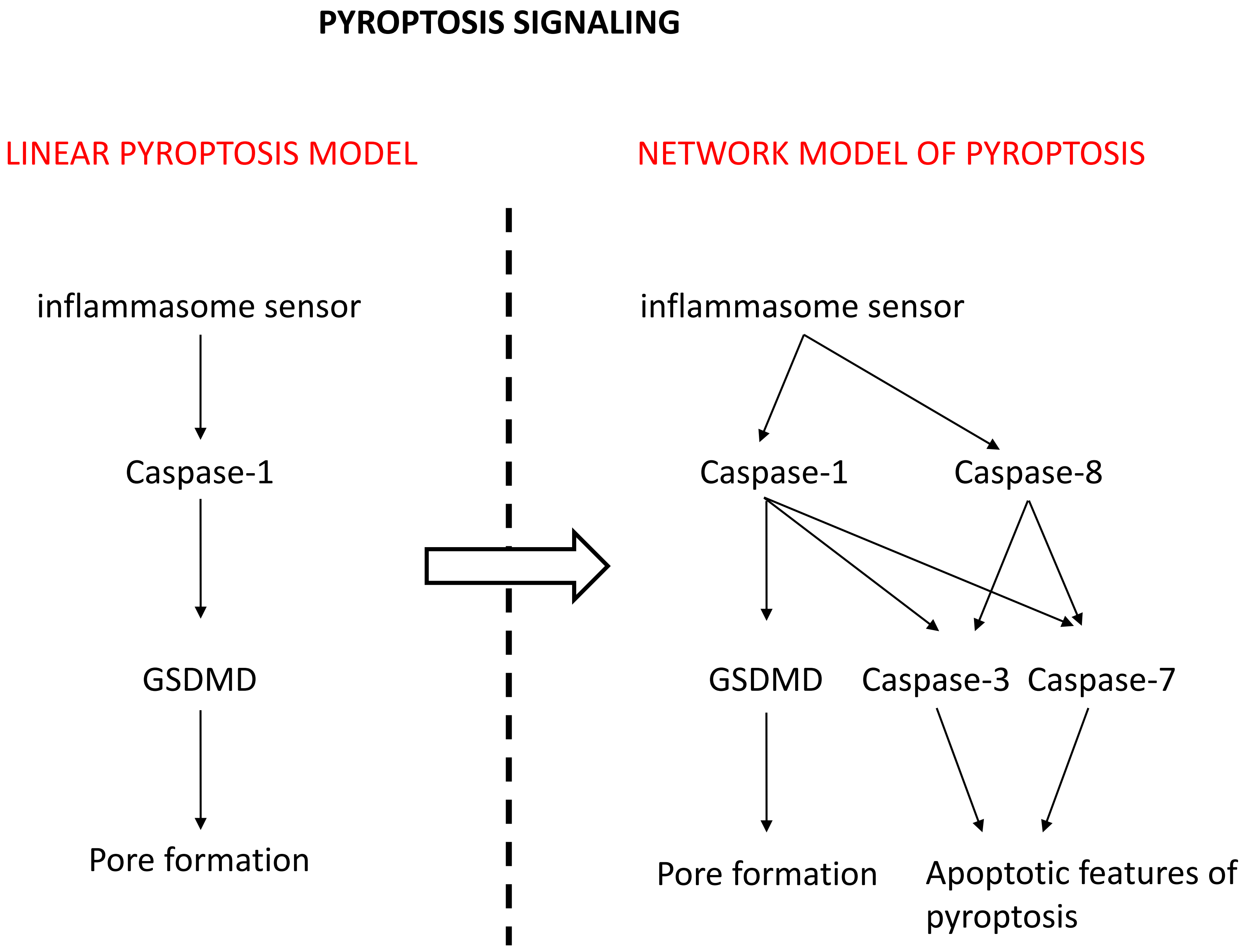
PYROPTOSIS SIGNALING
LINEAR PYROPTOSIS MODEL
NETWORK MODEL OF PYROPTOSIS
inflammasome sensor
Caspase-1
GSDMD
Pore formation
inflammasome sensor
Caspase-1
Caspase-8
GSDMD
Caspase-3
Caspase-7
Pore formation
Apoptotic features of pyroptosis

Fig. S5

Figure S5. Model for pyroptotic signaling network, related to Figures 1 and 6. Prior knowledge supported a linear model for pyroptosis induction, focused on how caspase-1 directed cleavage of GSDMD and release of a pore-forming N-terminal domain (on the right). Here, we demonstrate that both caspase-1 and caspase-8 act downstream of inflammasomes to initiate a caspase-3/-7 signature during pyroptosis, which is accompanied by cleavage of typically apoptotic markers, in parallel to caspase-1-initiated GSDMD cleavage and plasma membrane permeabilization (on the left).
